# Microstructure of Spark Plasma-Sintered Silicon Nitride Ceramics

**DOI:** 10.1186/s11671-017-2067-z

**Published:** 2017-04-24

**Authors:** O. A. Lukianova, V. Yu. Novikov, A. A. Parkhomenko, V. V. Sirota, V. V. Krasilnikov

**Affiliations:** 1 Belgorod National Research University, 85, Pobedy Str., 308015 Belgorod, Russia; 2 Institute of Solid State Physics, Materials Science and Technologies, NAS of Ukraine, Academic str. 1, Kharkov, 61108 Ukraine

**Keywords:** Silicon nitride, Spark plasma sintering, Microstructure, Hardness

## Abstract

The microstructure and phase composition of the high-content Al_2_O_3_-Y_2_O_3_-doped spark plasma-sintered silicon nitride were investigated. Fully dense silicon nitride ceramics with a typical α-Si_3_N_4_ equiaxed structure with average grain size from 200 to 530 nm, high elastic modulus of 288 GPa, and high hardness of 2038 HV were spark plasma sintered (SPSed) at 1550 °C. Silicon nitride with elongated β-Si_3_N_4_ grains, higher hardness of 1800 HV, density of 3.25 g/cm^3^, and Young’s modulus 300 GPa SPSed at 1650 °C was also reviewed.

## Background

Silicon nitride and SiAlON ceramics are promising engineering and structural ceramics due to their benefit combination of mechanical, thermal, and chemical properties. Silicon nitride ceramics can be successfully used for a wide range of applications where metals and polymers fail. For the manufacture of dense Si_3_N_4_, such sintering additives as aluminum oxide, yttrium oxide, or magnesium oxide are generally added to the original powder.

The most well-known commercial methods of Si_3_N_4_ production are a hot isostatic pressing (HIP) and spark plasma sintering (SPS). SPS which is one of the most innovative and promising methods of producing silicon nitride means sintering and sinter-bonding at low temperatures and short periods by charging the intervals between powder particles with electrical energy and effectively applying a high-temperature spark plasma. SPS systems offer many advantages over conventional systems using hot press (HP) sintering, HIP, or atmospheric furnaces, including ease of operation and accurate control of sintering energy as well as high sintering speed, high reproducibility, safety, and reliability. It is well known that an important role is played by the type of structure obtained by SPS. For instance, Peng have shown that using conventional hot pressing Y- and Yb-stabilized monophasic α-SiAlONs with stoichiometric composition retains the equiaxed morphology even at very high temperatures [1]. Peng also note that conditions suitable for elongated grain growth have never been established in such systems because of the lack of the formation and subsequent decomposition of intermediate phases during sintering. It should also be noted that the elongated morphology formation of structure is strongly controlled by such factors as a temperature and/or an extra liquid. However, there are two main points. First of all, in the absence of nitrogen overpressure, the silicon nitride dissociation begins above 1900 °C, and secondly, a large amount of liquid which later becomes the glass phase may reduce high-temperature properties of silicon nitride [1]. The spark plasma sintering is a modified method of hot pressing. SPS means a direct transmission of electric current directly through the mold and the pressed workpiece, and not through the external heater in comparison with the HP. Fast heating and short cycle times are achieved by a pulsed electric current and a so-called spark plasma effect.

In particular, these ceramics have high strength and relatively high fracture toughness, good wear resistance, and high oxidation resistance and corrosion resistance. Silicon nitride is being considered for a wide variety of structural application, such as the gas turbine engine, the next-generation power devices, turbocharger rotors, and diesel engine components [2–6].

The goal of this paper is to consider features of the microstructure and properties of the produced silicon nitride ceramics with a high content of yttrium and aluminum oxides. Spark plasma-sintered silicon nitride Al_2_O_3_-and-Y_2_O_3_-doped ceramics were selected as a material for the present investigation. The article refers also to free sintered silicon nitride Al_2_O_3_-Y_2_O_3_-doped ceramics.

## Methods

The starting Si_3_N_4_ powder was α-rich Si_3_N_4_ (Stark, Grade M11). Oxide-sintering additives were Y_2_O_3_ (Stark, Grade, 2 μm) and nanosized Al_2_O_3_ (A16 SG, 600 nm). Table 1 shows the initial ratio of the components. To assure a thorough homogeneity, the vibratory disc milling (Retsch RS-200) containing powder mixtures had also been performed for 20 min. The SPS process was carried out in a vacuum in a spark plasma-sintering apparatus, Dr. Sinter 2050 (Sumitomo Coal Mining Co. Ltd., Japan). The precursor powders were loaded in a cylindrical carbon die with an inner diameter of 20 mm. The samples were heated by passing a pulsed DC current through the pressurized die, i.e., using the die also as a heating source. The final sintering temperature was 1550 and 1650 °C with a 10-min holding time. The temperature was controlled by an optical pyrometer focused on the surface of the die. A pressure of 50 MPa was maintained throughout the sintering cycle.

**Table 1:** Chemical composition of the starting materials

Si_3_N_4_, wt%	Al_2_O_3_ wt%	Y_2_O_3_ wt%	Holding time, min
85	9	6	10

The phase composition of the sintered samples was determined by the X-ray diffraction method (XRD, diffractometer Rigaku Ultima IV; Cu Kα emission (radiation), Ni filter). A scan rate of 10 °C/min was used to record the diffraction patterns in 2*θ* range between 10 and 80 °C. XRD analyses were carried out using a Rigaku Ultima IV automated diffractometer. The sintered material was analyzed in the solid form.

For the Vickers indentations, the samples of approximately 60 mm-20 mm-30 mm in size were polished with diamond paste on a standard metallographic wheel, using 6- and 3-μm diamond pastes. Vickers indentations were made at 30-N loads using an automatic microhardness analysis system DM-8 and a loading time of 15 s. The indentation sizes were measured immediately after unloading. A total of 25 perfect indentations were made at each load with the Vickers indenter.

The microstructure was characterized by scanning electron microscopy (SEM). Structural characterization was performed using a Quanta 600 FEG (FEI company, Hillsboro, OR) scanning electron microscope. Since the silicon nitride material under investigation was non-conducting, it was necessary to coat it with a thin layer of carbon to prevent surface charging during examination.

Density of the samples was determined by a helium pycnometer (Micromeritics AccuPyc 1340). The indentation module (Young’s modulus) was measured using an automated Shimadzu DUH-211/DUH-211S.

## Results and Discussion

On the one hand, one of the most important advantages of ceramics is their low weight. But on the other hand, it is obvious that the high density and low porosity are key factors for many structural applications. For both sets of samples, the lack of porosity has been found (Table 2). The density of the described samples SPSed at 1550 °C was 3.21 g/cm^3^ by helium pycnometry, and it was 3.25 g/cm^3^ for samples SPSed at 1650 °C. All specimens were sintered to nearly full density (Table 2). It is obvious that the density of ceramics depends on porosity and on the pore size. Thus, ceramics produced by SPS and HIP methods have the highest density close to the theoretical one [6]. Belmonte et al. described silicon nitride ceramics obtained by SPS with a density of 3.08 g/cm^3^ sintered at 1500 °C and ceramics with a density of 3.23 g/cm^3^ sintered at 1600 °C and also ceramics sintered at 1650 °C with a density of 3.23 g/cm^3^, respectively [2]. Balázsi et al. described carbon nanotube-reinforced silicon nitride composites obtained by SPS with the density from 3.17 to 3.24 g/cm^3^ [7]. Hayashi et al. described ceramic material obtained by sintering in a graphite resistance furnace at 1900 °C for 2 to 48 h under a nitrogen pressure of 0.9 MPa with a density ranged from 3.20 to 3.26 g/cm^3^ with MgSiN_2_ as an additive and with a density ranged from 3.22 to 3.30 g/cm^3^ with MgO as an additive [4].

**Table 2:** Physical properties

SPS temperature, °C	*ρ*, g/cm^3^	Porosity
1550	3.21	–
1650	3.25	–

**Fig. 1:**
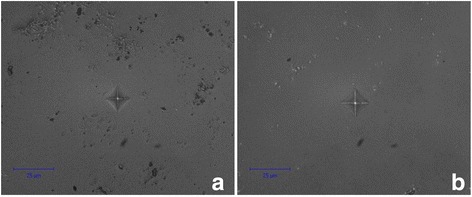
Vickers fingerprints of the SPSed Si_3_N_4_ with Al_2_O_3_-Y_2_O_3_ at **a** 1550 °C and **b** 1650 °C

The hardness of silicon nitride ceramics is high and linearly depends on the strength properties. Figure 1a, b illustrates two typical Vickers fingerprints. An indentation load has been chosen experimentally. The hardness of the obtained material was 2038 HV for ceramics SPSed at 1550 °C and 1800 HV for ceramics SPSed at 1650 °C (Table 3). For instance, the microhardness of the Si_3_N_4_ composite with 3 wt% CNT was 12 GPa (1224 HV) [8]. Silicon nitride ceramics a priori have high hardness especially for ceramics produced by such methods as HP, HIP, SN and SPS [9, 10]. It is also known that the hardness of α-Si_3_N_4_ is higher than the hardness of β-Si_3_N_4_ [1]. Balázsi et al. described the carbon nanotube-reinforced silicon nitride composites obtained by SPS with a microhardness from 1660 to 2010 HV [8].

**Table 3:** Mechanical properties

Sintering temperature, °C	*E*_IT_, GPa	Hardness, HV
1550	288	2038
1650	300	1800

The microhardness 16.6 GPa and the elastic modulus 285 GPa of the carbon nanotube-reinforced Si_3_N_4_ with 4 wt% Al_2_O_3_ and 6 wt% Y_2_O_3_ were significantly lower than without it [3].

**Fig. 2:**
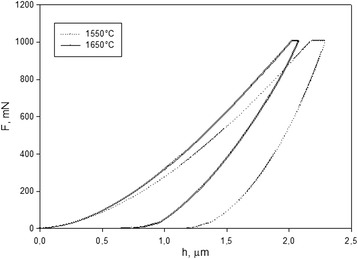
Load–displacement curves

The indentation modulus of the investigated material SPSed at 1550 °C was 288 GPa and 300 GPa for the material sintered at 1650 °C (Table 3). Load–displacement curves are shown in Fig. 2. Usually, the indentation modulus has the same value as Young’s modulus. For comparison, Young’s modulus for the same free sintered material was 240 GPa by resonance tests and 244 GPa by indentation modulus [11].

Young’s modulus of the HIPed silicon nitride was 320 GPa while Young’s modulus of the sintered silicon nitride was 290 GPa [2]. In our previous article, we described the silicon nitride pressureless sintered with Young’s modulus of 214 GPa [11]. Young’s modulus of spark plasma-sintered carbon nanotube-reinforced silicon nitride composites with 4 wt% Al_2_O_3_ and 6 wt% Y_2_O_3_ ranged from 286 to 326 GPa, and the shear modulus ranged from 115 to 130 GPa depending on the content of the multiwall carbon nanotubes [7].

Balázsi et al. described the HPed silicon nitride with low Young’s modulus of 150 GPa and low density compared with the density and Young’s modulus of the spark plasma-sintered silicon nitride [7, 8].

Shimada et al. described the high-temperature dependence of Young’s modulus with 3 wt% MgO fabricated by pressureless sintering in a nitrogen gas silicon nitride at 1750 °C. Young’s modulus decreased from 370 to 330 GPa with an increasing temperature up to 800 °C [12].

It is obvious that the phase composition clearly depend on such sintering parameters as the temperature of the sintering and the holding time on the one hand and the type and amount of the sintering agents on the other hand. Shen et al. described the Si_3_N_4_ ceramics SPSed at 1600 °C (holding time from 0 to 5 min) and sintered at 1700 °C. The α-Si_3_N_4_ and β-silicon nitride for pure α-Si_3_N_4_ powder without additives was observed. β-Si_3_N_4_ and β-SiAlON were observed at 1700 °C without holding time for initial β-SiAlON composition with AlN and Al_2_O_3_ additives. α-Si_3_N_4_ were observed after 5-min sintering at 1700 °C with high content of Yb_2_O_3_. However, β-silicon nitride and glass phase were observed at 1600 and 1700 °C without holding. Only β-Si_3_N_4_ and glass phase were observed after 5 min sintering at 1700 °C [13]. Perera et al. described the Si_3_N_4_/SiC composites SPSed at 1500 °C with a phase composition consisting of α-Si_3_N_4_ and β-Si_3_N_4_ and open porosity of 24%. Increase of the sintering temperature from 1650 to 1900 °C leads to increase of the β-phase content and decrease of the α-phase content [6]. Corral et al. described single-walled carbon nanotube (SWNT–Si_3_N_4_) nanocomposites processed using 1-, 2-, and 6-vol.% SWNTs and SPS [3].

Balázsi et al. showed a similar comparison with the described material with 10 wt% Al_2_O_3_ and Y_2_O_3_ oxide additives. A single-phase β-SiAlON nanoceramic Si_5_AlON_7_ has been prepared by high-energy mechanical milling and spark plasma sintering at 1550 °C for 5 min. An amorphous Si–C–N powder was used to obtain polycrystalline Si_3_N_4_/SiC ceramic composites. Optimal temperature for sintering was 1600 °C, and the primary phases were β-Si_3_N_4_ and β-SiC with approximately equal proportions of the two phases, along with minor phases such as silicon oxynitride, yttrium silicate, and some free carbon [8]. 

**Fig. 3:**
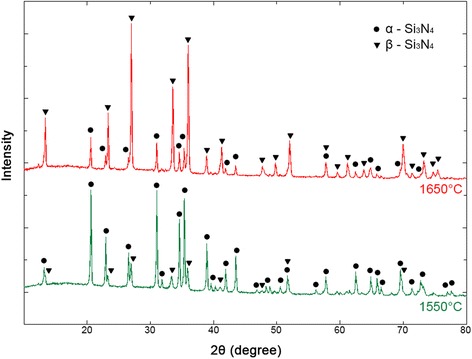
The X-ray analysis of the fabricated ceramics

Peng also show that aluminum-and-yttrium-doped spark plasma-sintered silicon nitride ceramics had a 59% un-reacted α-Si_3_N_4_, 13% β-SiAlON, and 28% α-SiAlON [1]. The described ceramic sample that had α-Si_3_N_4_ (76%) as a major phase and β-Si_3_N_4_ (24%) as a minor phase for ceramics was SPSed at 1550 °C, and conversely, the ceramic sample that had β-Si_3_N_4_ (72%) as a major phase and α-Si_3_N_4_ (28%) as a minor phase for ceramics was sintered at 1650 °C (Fig. 3 and Table 4). The quantitative phase composition of the produced ceramics and such characteristics as the crystal lattice parameters and the space symmetry group are given in Table 4. Balázsi et al. investigated samples SPSed for 3 min at 1500 °C and 50 MPa with main lines of α-Si_3_N_4_ and small additions of β-Si_3_N_4_ and multi-walled carbon nanotube (MWNT)–Si_3_N_4_ samples sintered by SPS for 5 min at 1500 °C and 100 MPa which showed main lines of α- and β-Si_3_N_4_. The sample sintered by SPS for 5 min at 1650 °C and 50 MPa demonstrated main lines of α-Si_3_N_4_ and β-Si_3_N_4_. The MWNT-doped Si_3_N_4_ sintered by SPS for 3 min at 1650 °C and 50 MPa was characterized by main lines of α-Si_3_N_4_ and small additions of β-Si_3_N_4_ [7]. The content of the α-Si_3_N_4_ phase changes from 76 to 6% depending on the sintering temperature of 1500 to 1650 °C, respectively [2].

**Table 4:** XRD and characteristics of the obtained phases

		The lattice constant, Å		Phase, %	
		a	c	1550	1650
β-Si_3_N_4_	P6_3_ (173)	7.629	2.927	24	72
α-Si_3_N_4_	P31c (159)	7.753	5.624	76	28

**Fig. 4:**
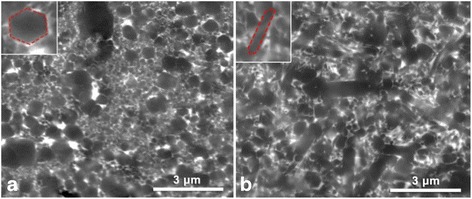
Microstructure of Si_3_N_4_ SPSed at **a** 1550 °C and **b** 1650 °C

The fully dense compacts obtained at 1550 °C consist of nearly equiaxed grains of sub-micron size. The typical α-Si_3_N_4_ ultrafine equiaxed hexagonal grained microstructures formed after SPS at 1550 °C. The average grain size is changed from 200 to 530 nm. By contrast, randomly selected ultrafine equiaxed and elongated β-grain microstructures developed by sintering at 1650 °C are fairly uniform. The glass phase at the grain boundaries and the lack of porosity were also observed for the both investigated ceramics (Fig. 4).

A grain-coarsening process (Fig. 4a in comparison to Fig. 4b), which can be directly attributed to thermally activated fast-growing process governed by dynamic Ostwald ripening, is observed. The grain growth is accompanied by α-β Si_3_N_4_ transformation in the sintering processes (Fig. 4). However, the influence of additives on the microstructure of similar material obtained by free sintering was described in our earlier work [14–17]. The negative effect of magnesium oxide on the structure of pressureless sintered ceramics was also shown earlier [17].

## Conclusions

In keeping with the results summarized above, the silicon nitride ceramic with a high content of sintering additives was produced by spark plasma sintering at 1550 and 1650 °C. The microstructure, phase composition, and mechanical and elastic properties of the produced ceramics were investigated. Proposed technology can be potentially applied in various fields of technology and industry in production of structural ceramics based on silicon nitride. By selecting appropriate compositions and sintering parameters, fully dense ceramics with tailored microstructures can be obtained, which consist of either submicron-sized grains with equiaxed morphology or well-facetted grains with elongated morphology. This opens up new possibilities for further materials development. It has been demonstrated that SPS is an efficient technique to implement this concept.

The results can be summarized as follows:

1. The density of the obtained material was in the narrow range from 3.21 to 3.25 g/cm^3^.
2. The hardness of the produced ceramics was 2038 HV for SPS at 1550 °C and 1800 HV for SPS at 1650 °C. The indentation modulus was 288 GPa for ceramics SPSed at 1550 °C and 300 GPa for material SPSed at 1650 °C.
3. The described ceramics SPSed at 1550 °C had α-Si_3_N_4_ as a major phase, and conversely, ceramics SPSed at 1650 °C had β-Si_3_N_4_ as a major phase.

## Abbreviations

CNT: Carbon nanotubes
HIP: Hot isostatic pressing
HP: Hot pressing
MWNT: Multi-walled carbon nanotubes
RFBR: Russian Foundation for Basic Research
SEM: Scanning electron microscopy
SN: Sintering
SPS: Spark plasma sintering
SWNT: Single-walled carbon nanotubes
XRD: X-ray diffraction
